# Digestive enzyme replacement relieves growth failure in preterm infants with poor exocrine pancreatic function: a retrospective case series

**DOI:** 10.1007/s00431-021-04069-0

**Published:** 2021-04-10

**Authors:** Annette Münch, Christoph Bührer, Ann Carolin Longardt

**Affiliations:** 1 Department of Neonatology, Charité - Universitätsmedizin Berlin, Berlin, Germany; 2 Department of Pediatrics, German Red Cross Hospital Westend, Berlin, Germany; 3 Children’s Hospital, Universitätsklinikum Schleswig-Holstein Campus Kiel, Kiel, Germany

**Keywords:** Growth failure, Exocrine pancreatic insufficiency, Very low birth weight infant, Enzyme replacement, Case series

## Abstract

In orally fed preterm infants, poor weight gain may be linked to low fecal pancreatic elastase-1 (FPE-1) activity, indicative of exocrine pancreatic insufficiency. The objective of this study was the retrospective assessment of the effect of exogenous digestive enzyme replacement by gavage in preterm infants with growth failure and low FPE-1 (<200 μg/g). We analyzed weight gain relative to baseline and caloric intake during 14-day periods before and after institution of digestive enzyme replacement containing 6000 U lipase and 240 U protease kg^−1^ d^−1^. Among 46 of 132 preterm infants < 1250g birth weight surviving to at least 14 days in whom FPE-1 was determined, 38 infants had low FPE-1 (< 200 μg/g), and 33 infants received exogenous digestive enzyme replacement. Average daily weight gain significantly increased from 14.4 [range 2.6–22.4] g kg^−1^ d^−1^ to 17.4 [8.4–29.0] g kg^−1^ d^−1^ (*P* = 0.001), as did weight gain per kcal, from 0.08 [0.02–0.13] g kcal^−1^ d^−1^ to 0.11 [0.05–0.18] g kcal^−1^ d^−1^.

*Conclusion*: In preterm infants with signs and symptoms of exocrine pancreatic insufficiency, exogenous digestive enzyme replacement is associated with improved growth.

**Table:** 

**What is Known:** *• Very preterm infants on full enteral nutrition may display growth failure linked to transient poor exocrine pancreatic function.* *• Porcine pancreatic enzymes covered with an acid-resistant coating are too large to pass the internal diameter of most gavage tubes used in very preterm infants.* **What is New:** *• Administration of a liquid formulation of acid-resistant microbial digestive enzymes in preterm infants with growth failure and low fecal pancreatic elastase-1 values was associated with improved weight gain.* *• Response to exogenous digestive enzyme replacement was associated with the prior extent of growth failure.*

**Supplementary Information:**

The online version contains supplementary material available at 10.1007/s00431-021-04069-0.

## Introduction

Postnatal growth failure and altered body composition of very preterm infants are associated with impaired neurodevelopmental outcome [1, 2]. Hence, current nutritional concepts aim to achieve intrauterine-like growth by commencement of parenteral nutrition on the first day of life and subsequent aggressive enteral nutrition to provide infants with adequate protein and energy intake [3]. A considerable proportion of very preterm infants, however, display poor growth [4], which may prompt the neonatologist to increase enteral supply. This approach may be limited by feeding intolerance and the need for fluid restriction in the presence of a patent ductus arteriosus or respiratory compromise. Moreover, increased supply of nutrients does not necessarily result in weight gain [5]. Furthermore, there are concerns about short-term complications, such as lactobezoars (milk curd syndrome) in the wake of extensive supplementation of breast milk with protein, calcium, and phosphorus [6] which creates an imbalance between enteral food supply and digestive capacity.

We have shown previously that a considerable proportion of very preterm infants display a protracted maturation of exocrine pancreatic function, as measured by fecal pancreatic elastase-1 (FPE-1) [7]. A retrospective cohort analysis of 26 preterm infants with poor postnatal growth despite aggressive enteral nutritional support found improved weight gain associated with administration of porcine pancreatic enzymes administered as acid-resistant microspheres [8]. However, removal of the acid-resistant coating by grounding of the microspheres (to enable their passage through the gavage tube) leads to inconsistent enzyme activities in the gut. This may be overcome by administration of a liquid solution containing digestive enzymes of microbial origin that are intrinsically acid resistant [9]. In the present study, we explored this approach in a cohort of very low birth weight infants with poor exocrine pancreatic function and postnatal growth failure despite high enteral food supply. We hypothesized that exogenous digestive enzyme replacement with this preparation is able to improve weight gain in very preterm infants with growth failure and poor exocrine pancreatic function.

## Methods

### Study design, setting, and patients

This retrospective study included all preterm infants born between March 01, 2017, and May 31, 2018, who were admitted to the Department of Neonatology at Charité - Universitätsmedizin Berlin, Germany, with a birth weight below 1250g and a gestational age below 32 weeks, who survived for more than 14 days, displayed postnatal growth failure despite intensified enteral nutritional support and exocrine pancreatic insufficiency (FPE-1 level < 200 μg/g), and were eventually treated with a water-soluble preparation of microbial digestive enzymes derived from *Rhizopus oryzae* and *Aspergillus oryzae* (Pankreafix, Rainfarn Pharmacy, München, Germany). The mixture of these enzymes (Nortase, Repha GmbH, Langenhagen, Germany) is licensed in Germany to treat exocrine pancreatic insufficiency without age restriction.

### Nutritional management

Infants were treated according to a standardized feeding protocol aimed to promote intrauterine-like growth following the respective fetal growth percentile values [10]. Fluids were started at 90 mL kg^−1^ d^−1^ and were incrementally increased to 160–180 mL kg^−1^ d^−1^ during 7–10 days. Parenteral nutrition, including amino acids and lipids, commenced on the first day of life and was maintained until the level of enteral feeding attained 130–150 mL kg^−1^ d^−1^. Enteral nutrition was started on the first day of life by 10 mL kg^−1^ d^−1^ and was increased daily whenever possible. Human milk, preferably the mother’s own breastmilk, was used. If the mother was tested positive for antibodies to cytomegalovirus, expressed breastmilk was pasteurized until the infant attained 32 weeks postmenstrual age or weight exceeded 1500 g. If no mother’s own breast milk was available, the infant received human donor milk after maternal consent. Human donor milk was always pasteurized. The protein content of human milk was increased by addition of bovine milk protein-based fortifiers (Aptamil FMS, Milupa, Friedrichsdorf, Germany, or Nestlé BEBA FM 85 Frauenmilchsupplement Pulver, Nestlé Deutschland, Frankfurt, Germany) with or without additional protein supplement (Aptamil Protein Plus, Milupa) when the daily volume exceeded 90 mL. The regular target protein intake was 4 (–4.5) g kg^−1.^d^−1^, and the target fluid intake on enteral nutrition was 160 (−180) mL kg^−1.^d^−1^.

### Postnatal growth failure

Anthropometric measurements were carried out regularly and plotted on the 2013 Fenton growth reference chart [10]. Weight was determined at least every 3 days, whereas length and head circumference were measured on a weekly basis. Several percentile curves were highlighted on the growth reference chart: the 3rd, 10th, 20th, 50th, 80th, 90th, and 97th percentile. Postnatal failure to thrive was assumed if weight gain diverged more than one percentile category from the desired growth curve (corresponding to a loss of more than 0.6 standard deviation scores of weight for 7 days without improvement) or an average daily weight gain below 1.5%.

For infants showing growth failure, the target energy intake of 130 kcal kg^−1^ d^−1^ was increased up to 150–165 kcal kg^−1^ d ^−1^. This was achieved by increasing fluid intake on enteral nutrition up to 200 mL^.^kg^−1^ d^−1^, supplementation of protein by bovine milk protein-based fortifiers up to 4.5–5 g kg^−1^ d^−1^ and/or addition of easily digested medium chain triglycerides (Ceres-MCT Öl®, Kanso, Dr. Schär AG, Burgstall (BZ), Italy) if tolerated by the neonate.

### FPE-1 measurements

FPE-1 concentrations were determined, at the discretion of the attending physician, at the age of 3–4 weeks if weight gain remained under the desired growth percentiles despite high energy intake. FPE-1 levels were measured after dilution (1:500) with an enzyme-linked immunosorbent assay (ScheBo; Biotech AG, Gießen, Germany) that employs two monoclonal antibodies reactive with chymotrypsin-like elastase 3A and 3B [11], with a detection limit of 1 ng/mL, an intra-assay variance of 5.8%, and an interassay variance of 7.7%. At a cut-off of <200 μg per gram of stool [12], reported sensitivities for mild, moderate, and severe pancreatic insufficiency in adult patients are 63%, 100%, and 100%, with a specificity of 93% [13]. This cut-off is likewise being used in newborn infants with cystic fibrosis to diagnose pancreatic insufficiency and start exogenous digestive enzyme replacement [14, 15].

### Exogenous digestive enzyme replacement

Digestive enzymes were prepared by adding 5000 U lipase from *Rhizopus oryzae*, 200 U protease and 3600 U amylase from *Aspergillus oryzae* [9] dissolved in 2 mL purified water to the milk feed directly before feeding via gavage. The total dose given was 6000 U lipase/4320 U amylase/240 U protease kg^−1^ d^−1^, divided into 8 or 12 doses. In case of persisting growth failure, doses were adjusted up to 12,000 U lipase/480 U protease kg^−1^ d^−1^. In the event of suspected side effects such as diaper dermatitis, the daily dosage was reduced or stopped. Otherwise, treatment was continued as long as a gavage tube was in place and used for most meals.

### Data retrieval

Medical charts of all infants were reviewed at three points of time: two weeks before starting digestive enzyme replacement, when digestive enzyme replacement commenced, and 14 days following the onset of digestive enzyme replacement. Data recorded were weight, length, head circumference, postmenstrual age, type and composition of nutrients (daily intake of energy, protein, carbohydrates, fat, and total fluids), and presence of diaper dermatitis.

### Statistical analysis

The primary outcome criterion was the development of growth velocity rates during the 14 days before and 14 days after onset of digestive enzyme replacement. Growth velocity (g kg^−1^ d^−1^) and daily weight gain per unit of energy intake (g kcal^−1^ d^−1^) were calculated exponentially according to Patel et al. [16]. Development of head circumference and length was analyzed on a weekly basis. Continuous data were presented as median and range or interquartile range and categorical data as percentage. The differences were compared by the paired Wilcoxon test. A *P* value < 0.05 was considered to indicate statistical significance. All analyses were performed employing SPSS 25.0 software (SPSS Inc, Chicago, IL).

## Results

### Patients’ characteristics

**Fig. 1:**
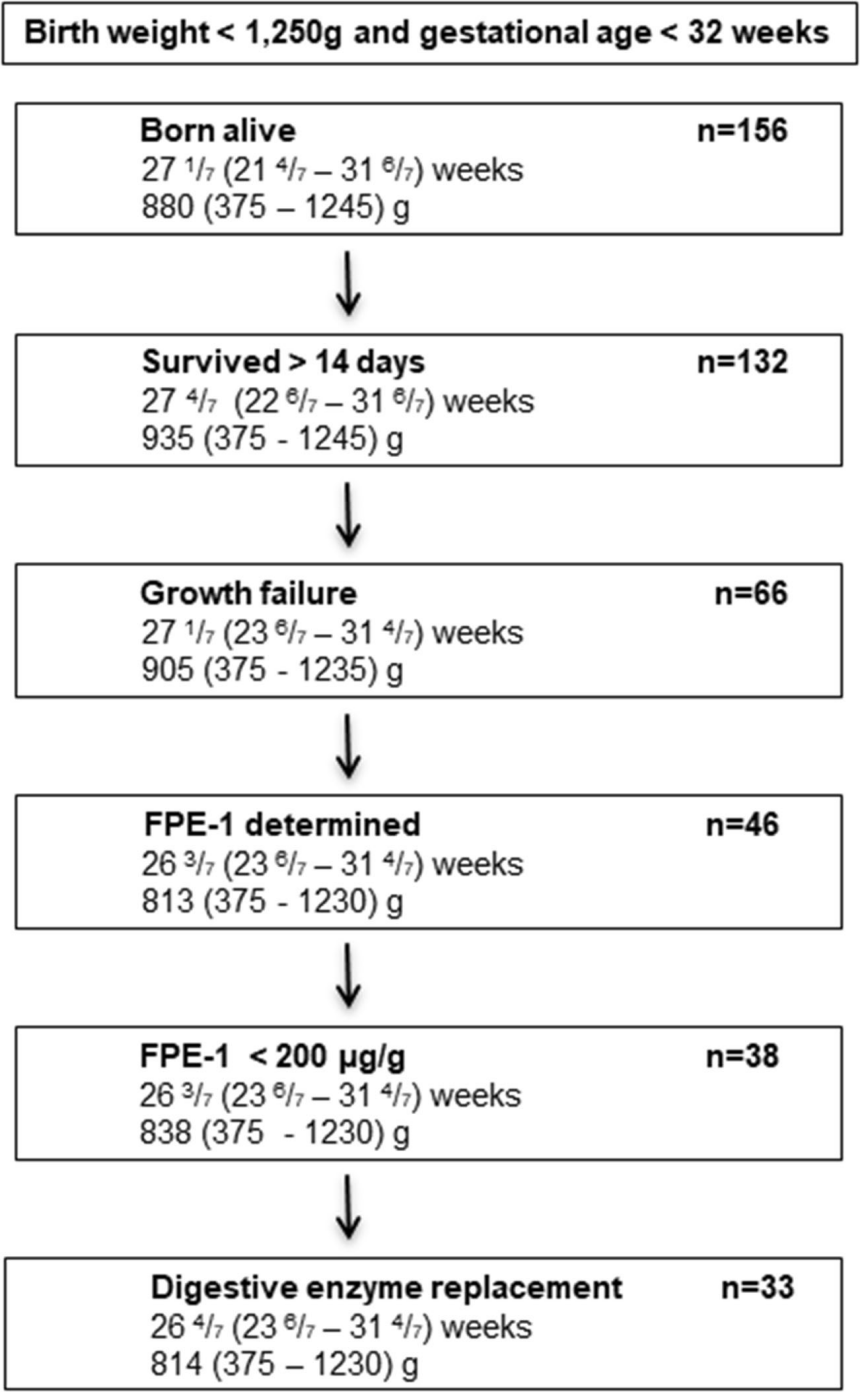
Flow chart of participants, with median and range of gestational age (weeks) and birth weight (g)

Between March 1, 2017, and May 31, 2018, 132 of 156 preterm infants admitted with a birth weight below 1250g and a gestational age below 32 weeks survived for more than 14 days, 66 of whom showed growth restriction. In 46 of these infants, FPE-1 levels were determined at least once (median age at first measurement 29 days, interquartile range 24–39 days), and 38 had FPE-1 levels < 200 μg/g. Out of these, 33 infants received exogenous digestive enzyme replacement at the discretion of the attending physician (Fig. 1).

Clinical characteristics of patients receiving digestive enzyme replacement are shown in Table 1. The nutritional constituents are depicted in Table 2. Two weeks before starting digestive enzyme replacement (*T*_0_-14 d), a minor proportion of patients (30%) had an intravenous line and received some parenteral nutrition, but all patients were on full enteral feeds when digestive enzyme replacement was started (*T*_0_). Nutrient intakes were increased during the 14 days before starting enzyme replacement, whereas during the treatment period (*T*_0_ – *T*_0_+14 d), calculated nutrient intake rather declined (Table 2).

**Table 1:** Clinical characteristics of patients receiving exogenous digestive enzyme replacement

Number of patients	33 (100%)
Female/male	21 (64 %)
Birth weight (g)	855 (375–1230)
Small for gestational age (< 10th percentile)	7 (21 %)
Gestational age (weeks)	26 ^0^/_7_ (23 ^0^/_7_–31 ^0^/_7_)
CRIB (clinical risk index for babies)	6 (1–13)
Supplemental oxygen requirement at 36-week postmenstrual age	4 (12 %)
Patent ductus arteriosus treated with ibuprofen	7 (21 %)
Retinopathy of prematurity treated with bevacizumab	2 (6 %)
Intraventricular hemorrhage	4 (12%)
Antibiotics (any time *t*_0_ – 14 d until *t*_0_ +14 d)	12 (36 %)
Fecal pancreatic elastase-1 (μg/g)	88 (31–187)
Duration of exogenous digestive enzyme replacement (days)	27 (5–60)

Data are presented as numbers (percentage) of subjects or median (range)

**Table 2:** Details of weight and nutritional intake 14 days before initiation of digestive enzyme replacement (*T*_0_– 14 d), when digestive enzyme replacement was started (*T*_0_), and 14 days after initiation of digestive enzyme replacement (*T*_0_ + 14 d)

	*T*_0_ - 14 d	*T*_0_	*T*_0_ +14 d
Number of patients	33 (100 %)	33 (100 %)	33 (100 %)
Actual weight (g)	1020 (670–2335)	1220 (815–2420)	1550 (1100–2720)
Nutrition^b^			
Carbohydrates (g kg^−1^ d ^−1^)	14.4 (10.5–19)	16.1 (12.7–19.9)	15.5 (11.7–18.1)
Protein (g kg^−1^ d^−1^)	4.4 (2.5–5.1)	4.5 (3.8–5.2)	4.3 (3.0–5.6)
Lipids (g kg^−1^ d^−1^)	7.3 (4.8–11.4)	9.6 (6.9–17.8)	8.9 (6.8–12.6)
Fluids (mL kg^−1^ d^−1^)	176 (154–273)	187 (161–216)	187 (159–220)
No intravenous line	23 (70 %)	33 (100 %)	32 (97 %)
Caloric intake (kcal kg^−1^ d^−1^)	140 (108–179)	161 (138–190)	153 (130–197)

Data are presented as numbers (percentage) of subjects or median (range) of continuous variables

### Effects of digestive enzyme replacement on weight gain and head circumference

Infants receiving digestive enzyme replacement showed a significant increase of average daily weight gain (g kg^−1^ d^−1)^ and weight gain per unit of energy intake (g kcal^−1^ d^−1^) (Table 3). Average daily weight gain was significantly lower in the pre-substitutional time period (median [range]) 14.4 [2.6–22.4] g kg^−1^ d^−1^ than in the substitutional period (17.4 [8.4–29.0] g kg^−1^ d^−1^ (*P* = 0.001). A similar picture emerged when calculating weight gain per unit of energy intake (*P* < 0.001) (Table 3). Growth rates for head circumference increased significantly (*P* = 0.028), while no effect was noted for body length.

**Table 3:** Effects of digestive enzyme replacement

	Before	During	*P* value
	digestive enzyme replacement		
Average daily weight gain (g^.^kg^−1.^d^−1^)			
All patients (*n*=33)	13.8 (2.6–22.4)	17.4 (8.4–29.0)	0.001
14 d before start of digestive enzyme replacement:			
Patients with intravenous line (*n*=10)	12.3 (6.3–18.2)	18.4 (15.2–23.5)	0.005
Patients on full enteral feeds (*n*=23)	14.4 (2.6–22.4)	17.0 (8.4–29.0)	0.073
Patients with very low (<100 μg/g) FPE-1 (*n*=17)	14.4 (6.2–21.6)	16.1 (11.7–29.0)	0.025
Patients with low (100–199 μg/g) FPE-1 (*n*=16)	14.2 (2.6–22.4)	17.7 (8.4–24.8)	0.017
Average daily weight gain per unit of energy intake (g^.^kcal^−1.^d^−1^)			
All patients (*n*=33)	0.08 (0.02–0.13)	0.10 (0.05–0.18)	< 0.001
14 d before start of digestive enzyme replacement:			
Patients with intravenous line (*n*=10)	0.07 (0.04–0.10)	0.11 (0.09–0.15)	0.005
Patients on full enteral feeds (*n*=23)	0.09 (0.02–0.13)	0.11 (0.05–0.13)	0.031
Patients with very low (<100 μg/g) FPE-1 (*n*=17)	0.09 (0.03–0.12)	0.10 (0.06–0.18)	0.013
Patients with low (100–199 μg/g) FPE-1 (*n*=16)	0.08 (0.02–0.13)	0.11 (0.05–0.17)	0.013
Head circumference (cm^.^week^−1^)	0.74 (−0.25–1.45)	0.95 (0.5–1.75)	0.028
Body length (cm^.^week^−1^)	1.2 (0–3)	1.3 (0.3–3.75)	0.896

Sensitivity analyses demonstrated that average daily weight gain per unit of energy intake increased significantly in all infants with poor growth, regardless of whether they received prolonged parenteral nutrition (*P* = 0.005) or not (*P* = 0.031), but the positive effect was more pronounced in infants who had been on supplemental intravenous nutrition 14 d before institution of exogenous digestive enzyme replacement (Table 3). At birth, these infants did not differ from infants fed exclusively enterally 14 days before starting exogenous digestive enzymes (similar birth weight, gestational age, and birth weight percentile) but had significantly lower weight percentiles at commencement of digestive enzyme replacement (6 [<1–23] vs 18 [1-32]). Average daily weight gain increased significantly in infants with poor growth (average daily weight gain less than 15 g kg^−1^ d^−1^) (*P* < 0.001), while there was no overall statistically significant increase in infants with an average daily weight gain exceeding 15 g kg ^−1^ d^−1^ during the last 14 days before institution of exogenous digestive enzyme replacement. An increase in weight gain in response to digestive enzyme replacement was both noted in infants with very low FPE-1 (< 100 μg/g) and in infants with low FPE-1 (100–199 μg/g).

FPE-1 was determined for a second time in 21 infants after a median interval of 22 days (interquartile range 16–27 days). FPE-1 increased in 19 of these infants (90%), with 5 infants (24%) displaying second FPE-1 levels exceeding 200 μg/g.

### Adverse events

All patients were monitored for signs of mucous membrane damage. Minor diaper dermatitis was present before initiation of digestive enzyme replacement in 3 (8.5%) infants and in 8 (23%) infants after 14 days of treatment (*P* > 0.1). The dosage was reduced in one patient after 8 days upon discretion of the attending physician because of diarrhea. Enzyme replacement was electively discontinued when infants no longer required a gavage tube in all but one patient who had digestive enzyme replacement withdrawn after 55 days for suspected gastroesophageal reflux and esophagitis.

## Discussion

In this cohort of preterm infants < 1250 g birth weight with growth failure and poor exocrine pancreatic function, weight gain and growth of head circumference were significantly improved during the 2 weeks following administration of digestive enzymes available for treatment of patients including infants with exocrine pancreatic insufficiency. Possibly related adverse effects were minor and reversible upon discontinuation of the digestive enzymes.

Microbial digestive enzyme replacement was initiated in a small cohort of preterm infants displaying signs and symptoms of transient poor pancreatic function. The decision to use a liquid formulation containing enzymes of microbial origin was made due to the lack of beneficial effects of porcine pancreatic enzyme replacement we observed when using ground microspheres of a commercially available product [7]. Grinding of the microspheres facilitates their passage through the gavage tube but may destroy their acid-resistant coating. The enzymes are therefore exposed to the acidic milieu of the stomach which promotes their inactivation. A relevant increase in weight gain of preterm infants has been reported during the initial 7 days following the initiation of the same exogenous pancreatic enzymes but without grinding the microspheres [8]. In our experience, this approach is limited by the narrow internal diameter of gastric tubes used in very preterm infants (1.2 mm, whereas the smallest diameter of the microspheres is 1.25 mm) and the inability of the preterm infants to swallow the microspheres. The preparation of microbial digestive enzymes consists of a water-soluble powder which is dissolved directly before administration, yielding a liquid that can pass easily through the gastric tube. The enzymes are licensed to treat patients with exocrine pancreatic insufficiency without age restriction and commonly used in newborn infants with cystic fibrosis detected by neonatal screening.

Another benefit might be that the acid-resistant microbial digestive enzymes used in this study, as opposed to porcine pancreatic enzymes, have a broader pH activity spectrum (pH 3-9) than porcine pancreatic enzymes (pH 5-7). The gastric content of preterm neonates becomes acidic on the first day of life, and a pH of 4–5 is maintained throughout the first month of life [17]. Moreover, enzymes of microbial origin, as opposed to those originating from slaughtered pigs, are likely more acceptable to vegans, Muslims, and observant Jews.

Skin or mucosal lesions may be unintended side effects of proteases given orally. We limited administration of the microbial digestive enzymes to infants with gavage tubes, and exogenous enzyme replacement was stopped prior to discharge in all cases. Given the short treatment period and the small number of infants, detection of rare side effects would have been impossible within the framework of this pilot study.

In the cohort studied here, the daily weight gain of some infants who originally met criteria for growth failure approached the desired target range before starting exogenous digestive enzyme replacement, possibly reflecting the maturation of exocrine pancreatic function [7]. These infants did not show consistent weight gain following exogenous digestive enzyme replacement. Similar to observations in a previous report using porcine pancreatic enzymes [8], poor growth rather than the extent of exocrine pancreatic insufficiency, as quantitated by FPE-1 levels in a spot stool sample, predicted response to exogenous digestive enzyme replacement. Of note, no statistically significant in growth velocity has been observed in a randomized controlled trial assigning unselected preterm infants (both with and without growth failure) to recombinant bile-salt-stimulated lipase [18].

The study presented here has several limitations. Notably, this was not a case-control study or a randomized controlled trial, and all data were collected in retrospect. It is not possible to draw a definite conclusion due to confounding factors including increasing gestational age. The sample size was limited as only a minor proportion of infants had both growth failure and laboratory signs of exocrine pancreatic insufficiency. Cessation of enzyme replacement mostly occurred at a time when infants became predominantly breastfed and often were discharged soon afterwards, precluding an assessment of how cessation of enzyme replacement affected growth patterns. Exogenous enzyme replacement was not assessed in infants with poor growth but FPE-1 levels >200 μg/g as these infants did not meet diagnostic criteria of exocrine pancreatic insufficiency. While the results must be considered preliminary, they may inform further research and provide data to estimate the sample size of a future randomized placebo-controlled prospective trial.

## Conclusion

Exogenous digestive enzyme replacement was associated with increased weight gain and head circumference growth in this cohort of preterm infants with signs and symptoms of exocrine pancreatic insufficiency. The effects were both statistically significant and clinically important for the infants concerned and warrant further investigations.

## Supplementary Information

ESM 1 (DOCX 14 kb)

## Abbreviations

FPE-1: Fecal pancreatic elastase-1
U: Unit
