# Supplementary material for: Digestive enzyme replacement relieves growth failure in preterm infants with poor exocrine pancreatic function: a retrospective case series
Source: Eur J Pediatr. 2021 Apr 10;180(9):2951–8. doi: 10.1007/s00431-021-04069-0 (PMC8346403; doi:10.1007/s00431-021-04069-0)
Supplement: Supplementary file 1 — (DOCX 14 kb) [file 431_2021_4069_MOESM1_ESM.docx]

**CARE** Checklist

Title

1 The diagnosis or intervention of primary focus followed by the words “case series” *Line 2*

Key Words

2 - 2 to 5 key words that identify diagnoses or interventions in this case report, including "case report" . . .  *Line 31*

Abstract (no references)

3a Introduction: What is unique about this case and what does it add to the scientific literature?

*Line 14-16*

3b Main symptoms and/or important clinical findings . . . . . . . . . . . . . . . . . . . . . . . ----- *Line 22*

3c The main diagnoses, therapeutic interventions, and outcomes . . . . . . . . . . . . . . . .*Lines 21-25*

3d Conclusion—What is the main “take-away” lesson(s) from this case? . . . . . . . . . . *Lines 26-27*

Introduction

4 One or two paragraphs summarizing why this case is unique (may include references) .

*n.a.,* *the cases are not unique but apparently comprise up to 25% of very preterm infants*

Patient Information

5a De-identified patient specific information. . . . . . . . . . . . . . . . . . . *Lines 159-167, Fig 1, Table 1*

5b Primary concerns and symptoms of the patient. . . . . . . . . . . . . . . . . . . *Lines 167-170, Table 2*

5c Medical, family, and psycho-social history including relevant genetic information . . . . . . . *n.a.*

5d Relevant past interventions with outcomes . . . . . . . . . . . . . . . . . . . . . . *Lines 173-204, Table 3*

Clinical Findings

6 Describe significant physical examination (PE) and important clinical findings. . . . *Table 1-3*

Timeline

7 Historical and current information from this episode of care organized as a timeline . . . *n.a.*

Diagnostic Assessment

8a Diagnostic testing (such as PE, laboratory testing, imaging, surveys). . . . . . . . . . *Lines 122-131*

8b Diagnostic challenges (such as access to testing, financial, or cultural) . . . . . . . . . . . . *n.a.*

8c Diagnosis (including other diagnoses considered) . . . . . . . . . . . . . . . . . . . . . . . . . . . . . *n.a.*

8d Prognosis (such as staging in oncology) where applicable . . . . . . . . . . . . . . . . . . . . . . . *n.a.*

Therapeutic Intervention

9a Types of therapeutic intervention (such as pharmacologic, surgical, preventive, self-care) . . .

9b Administration of therapeutic intervention (such as dosage, strength, duration) *Lines 133-40*

9c Changes in therapeutic intervention (with rationale) . . . . . . . . . . . . . . . . . . . . . . . . . . . .*Table 2*

Follow-up and Outcomes

10a Clinician and patient-assessed outcomes (if available) . . . . . . . . . . . . . . . . . . . . . . .. . . *Table 3*

10b Important follow-up diagnostic and other test results . . . . . . . . . . . . .*Table 3, Lines 194-196*

10c Intervention adherence and tolerability (How was this assessed?) . . . . . . . . . *Lines 198-204*

10d Adverse and unanticipated events . . . . . . . . . . . . . . . . . . . . . . . . . . . . . . . . . . . *Lines 198-204*

Discussion

11a A scientific discussion of the strengths AND limitations associated with this case report

*Lines 227-255*

11b Discussion of the relevant medical literature with references. . . . . . . . . . . . . .*Lines 212-246*

11c The scientific rationale for any conclusions (including assessment of possible causes)

*Lines 247-257*

11d The primary “take-away” lessons of this case report (without references) in a one paragraph conclusion . . . . . . . *Lines 259-263*

Patient Perspective

12 The patient should share their perspective in one to two paragraphs on the treatment(s) they received n.a.

Informed Consent

13 Did the patient give informed consent? Please provide if requested (Yes/No)…*Lines 275-276*
